# The attitudes, beliefs and behaviours of GPs regarding exercise for chronic knee pain: a systematic review

**DOI:** 10.1186/1471-2296-11-4

**Published:** 2010-01-18

**Authors:** Elizabeth Cottrell, Edward Roddy, Nadine E Foster

**Affiliations:** 1 Arthritis Research Campaign National Primary Care Centre, Primary Care Sciences, Keele University, Keele, Newcastle-under-Lyme, UK

## Abstract

**Background:**

Joint pain, specifically chronic knee pain (CKP), is a frequent cause of chronic pain and limitation of function and mobility among older adults. Multiple evidence-based guidelines recommend exercise as a first-line treatment for all patients with CKP or knee osteoarthritis (KOA), yet healthcare practitioners' attitudes and beliefs may limit their implementation. This systematic review aims to identify the attitudes, beliefs and behaviours of General Practitioners (GPs) regarding the use of exercise for CKP/KOA.

**Methods:**

We searched four electronic databases between inception and January 2008, using subject headings to identify studies examining the attitudes, beliefs or behaviours of GPs regarding the use of exercise for the treatment of CKP/KOA in adults aged over 45 years in primary care. Studies referring to patellofemoral pain syndrome or CKP secondary to other causes or that occurring in a prosthetic joint were excluded. Once inclusion and exclusion criteria were applied, study data were extracted and summarised. Study quality was independently reviewed using two assessment tools.

**Results:**

From 2135 potentially relevant articles, 20 were suitable for inclusion. A variety of study methodologies and approaches to measuring attitudes beliefs and behaviours were used among the studies. Quality assessment revealed good reporting of study objective, type, outcome factors and, generally, the sampling frame. However, criticisms included use of small sample sizes, low response rates and under-reporting of non-responder factors. Although 99% of GPs agreed that exercise should be used for CKP/KOA and reported ever providing advice or referring to a physiotherapist, up to 29% believed that rest was the optimum management approach. The frequency of actual provision of exercise advice or physiotherapy referral was lower. Estimates of provision of exercise advice and physiotherapy referral were generally higher for vignette-based studies (exercise advice 9%-89%; physiotherapy referral 44%-77%) than reviews of actual practice (exercise advice 5%-52%; physiotherapy referral 13-63%). *Advice to exercise *and exercise *prescription *were not clearly differentiated.

**Conclusions:**

Attitudes and beliefs of GPs towards exercise for CKP/KOA vary widely and exercise appears to be underused in the management of CKP/KOA. Limitations of the evidence base include the paucity of studies directly examining attitudes of GPs, poor methodological quality, limited generalisability of results and ambiguity concerning GPs' expected roles. Further investigation is required of the roles of GPs in using exercise as first-line management of CKP/KOA.

## Background

Joint pain, specifically chronic knee pain (CKP), is a frequent cause of primary care consultations and limitation of function and mobility among older adults. Approximately 25% of adults aged over 45 years have previously experienced knee pain lasting over a month or had an episode of knee pain in the last year and prevalence increases with age [1-3]. More than 90% of GPs manage at least one patient with severe knee pain over a two-week period [4]. In the UK, the National Institute for Health and Clinical Excellence (NICE) has recognised the importance of good management of peripheral arthritis by publishing guidelines, *Osteoarthritis: the care and management of osteoarthritis in older adults*, in February 2008 [5].

Evidence suggests that exercise improves functioning and symptoms in CKP/KOA [6] and has the supplementary benefits of improved cardiovascular status [7], emotional wellbeing [7] and proprioception [6]. Multiple generic, secondary and primary care guidelines recommend management for CKP and/or knee osteoarthritis (KOA) [6,8-13]. These, and a Cochrane review [14], conclude that exercise is beneficial and should be a first-line management strategy for CKP/KOA [5,6,12,15,16].

Providing advice to exercise will not necessarily improve patient outcomes. Patients must translate advice into action. They must follow advice correctly, for adequate time and with adequate intensity to improve function and symptoms. Patients may undertake exercise independently from GPs or other healthcare practitioners' advice [17]. Without instruction, motivated patients may exercise with little or no benefit [18].

As CKP/KOA is commonly managed within primary care, it is logical that GPs should implement guideline recommendations and advise patients to adopt and maintain exercise activity. GPs may *advise *patients to exercise, *prescribe *specific exercises of particular type, duration or frequency, or *refer *patients to another professional, for example a physiotherapist.

The implementation of guideline recommendations about exercise as a core management strategy for CKP/KOA may be influenced by the attitudes and beliefs of GPs regarding the use of exercise for this patient population. However, the nature of such attitudes/beliefs and the extent to which GPs recommend or use exercise for CKP/KOA is uncertain. A systematic literature review was conducted to investigate the attitudes, beliefs and behaviours of GPs, regarding exercise for CKP/KOA in adults aged 45 years or older.

## Methods

Search terms were chosen to identify research studies pertaining to CKP/KOA, exercise, GPs, attitudes or beliefs and behaviours, see Table 1. EC searched the databases MEDLINE, EMBASE, PsycINFO and CINAHL. Search terms were exploded and titles and abstracts were searched within articles from the database inception date to January 2008. Duplicates were removed. Title and abstracts of identified articles were reviewed. Articles failing to meet inclusion criteria and/or meeting at least one exclusion criterion were excluded. The full text of all remaining articles was reviewed, exclusion and inclusion criteria reapplied and non-relevant papers discarded. Additional relevant papers were sought from reference lists during full text review and from research team members who had identified them in previous CKP research. ER and NF independently reviewed the eligible literature for study inclusion. Where needed, authors were contacted to clarify/request data. Relevant papers published in non-English languages were translated.

**Table 1:** Search terms used


**Criteria**	**Search terms used (each term within criteria combined with Boolean Operator "OR")**

Chronic Knee Pain (CKP)	"Chronic knee pain"; "Chronic pain" AND "knee" OR "knee joint"; "Knee arthritis"; "Knee" OR "knee joint" AND "pain measurement"; "Knee osteoarthritis"; "Knee pain"; "Musculoskeletal pain"; "Osteoarthritis, knee"; "Osteoarthritis" AND "knee" OR "knee joint"; "Pain" AND "knee" OR "knee joint"

Exercise (Ex)	"Dance therapy"; "Dynamic exercise"; "Exercise"; "Exercise therapy"; "Motion therapy"; "Motor activity"; "Movement therapy"; "Muscle stretching exercises"; "Physical activity"; "Static exercise"; "Tai Chi"; "Therapeutic exercise"; "Walking"; "Yoga"

General practitioners (GP)	"Family medicine"; "Family physicians"; "Family practice"; "General practitioner"; "General practice"; "Physicians, family"; "Primary care"; "Primary health care"; "Primary" AND "healthcare"; "Primary medical care"; "Primary healthcare"

Attitudes, beliefs (At)	"Attitude" OR "Attitudes"; "Attitude of health personnel"; "Belief" OR "Beliefs"; "Health personnel attitude"; "Perception" OR "perceptions"; "Physician attitude"

Behaviours (Be)	"Adherence to guidelines"; "Approaches"; "Behaviours"; "Clinical practice"; "Case management"; "Disease management"; "Management"; "Medical treatment"; "Medical audit"; "Medical Practice"; "Pain management"; "Physicians Practice patterns"; "Prescription"; "Treatment Orientations"


### Quality assessment

All relevant studies were independently quality assessed by EC and either NF or ER using The Newcastle Critical Appraisal Worksheet (NCAW) [19], designed for any study type, and the Critical Appraisal Skills Programme (CASP) Qualitative Research Assessment Tool, designed for qualitative studies [20]. Disagreements were resolved through discussion by the initial assessors or using a third assessor.

### Inclusion criteria

Articles were relevant if they were empirical studies about knee pain, specifically CKP/KOA in adults over 45 years; related to primary care, included information about exercise and contained details about the attitudes, beliefs and/or behaviours of GPs towards exercise for CKP/KOA. There was no limit on research methodology or language of the original article. For this review, a working definition of CKP was mechanical knee pain, with or without loss of function, and with or without radiographic changes consistent with KOA, that has lasted for at least three months. Radiographic confirmation of KOA was not required due to the discordance between pain and OA-related radiographic changes [21].

### Exclusion criteria

Studies were excluded if they referred to patellofemoral pain syndrome alone, or CKP/KOA resulting from trauma, malignancy, infection, inflammatory arthritis or secondary to other diseases, or that occurring in a prosthetic joint.

### Attitude, beliefs and behaviour

The constructs of attitudes, beliefs and behaviours are complex. Therefore, for the purpose of this study, the following simplified working definitions were agreed among the authors and used. An attitude is defined as "a settled way of thinking" [22]. A belief is "an acceptance that something exists or is true" [22] or "a firmly held opinion or conviction" [22]. Attitudes and beliefs may be reported by study participants; alternatively they may be implied by physician behaviour. We reviewed reported and observed behaviours described by each study and considered whether these implied positive or negative attitudes and/or beliefs to exercise. For example, if a physician suggested rest for CKP/KOA in a study, the data was extracted as an implied belief that exercise for CKP/KOA would not be positive for the patient. Both implied and reported attitudes and beliefs were included and highlighted as such.

Behaviours are the ways in which one acts or conducts oneself [22]. Behaviours can result from attitudes and beliefs but may not truly indicate these. Behaviours can be reported or observed. Physician self-reported clinical management constitutes "reported behaviour". Data on "actual behaviour" refers to that which has either been collected through direct observation, patient report or from case-note or medical record review.

### Advice to, or prescription of, exercise?

The distinction between prescribing and advising exercise was defined by the amount and type of information relayed to the patient. To "*prescribe*" exercise, GPs should inform patients of the required type, duration and frequency of exercise. Exercise "*advice*" implies that the GP has recommended the patient to exercise and may have provided broad categories of exercise to undertake. GPs may "prescribe" or "advise" exercise through *referral *to a physiotherapist. Provision of an exercise leaflet is an easy and reproducible way of GPs providing consistent information to patients. However, leaflet provision may be considered to be either advice to exercise or prescription of exercise depending upon the information contained within it. Exercise prescription, however, requires information regarding the type, duration and suggested frequency of exercise which are likely to vary, at least initially, from patient to patient. Therefore, as a leaflet can only provide general, rather than patient-specific, advice, information leaflets were classified as advice to exercise rather than an exercise prescription for the purposes of this study.

## Results

### Literature search

**Figure 1:**
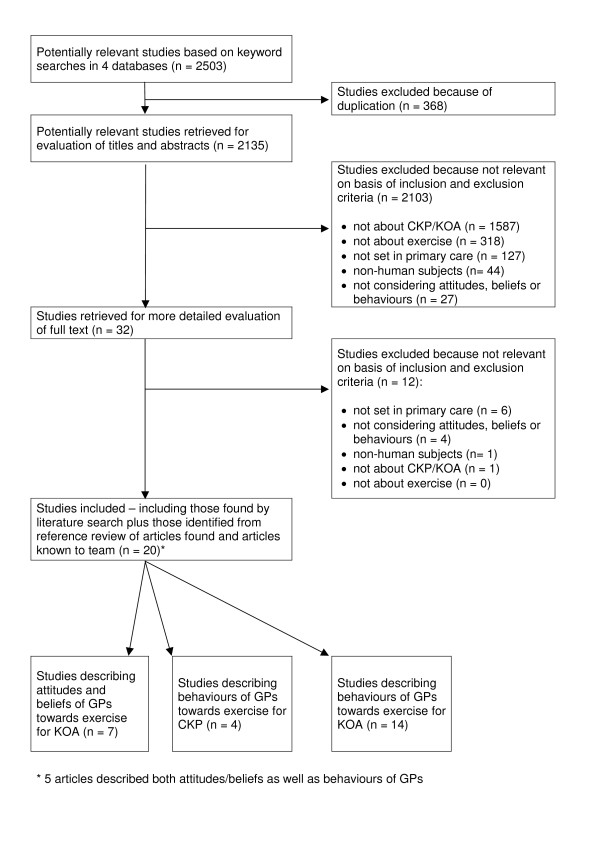
**Flow chart demonstrating results of literature search for attitudes, beliefs and behaviours of GPs about exercise for CKP/KOA**.

After removal of duplicates, 2135 articles were identified. Twenty papers reporting 20 different studies undertaken between 1992 and 2007 fulfilled the inclusion and exclusion criteria (Figure 1). Five articles described both attitudes and beliefs as well as behaviours of GPs, therefore of the 20 relevant articles, seven described attitudes and beliefs of GPs towards exercise for KOA [23-29] [Additional File 1] and eighteen described behaviours of GPs regarding exercise for CKP/KOA [4,23,27,30-42] [Additional File 2].

Of the 20 papers, three focussed on the management of patients with CKP, 16 focussed on patients with KOA, symptoms of KOA or clinical diagnosis of KOA, and one differentiated between CKP and KOA. The four studies that related specifically to CKP [30,35,38,40] were UK studies. Definitions of CKP and KOA used by many studies were unclear and/or inconsistent.

Of the studies investigating attitudes and beliefs of GPs towards exercise for KOA, one was performed in the UK, one in the Netherlands, two in Canada and three in France. One of the latter three also included practitioners from Belgium, Italy, Spain and Switzerland. Seven of the 18 studies investigating the behaviour of GPs regarding exercise for CKP/KOA were conducted in the UK. Of the remaining studies, two were from France, three from USA, two from Canada and one each from Netherlands, Germany, Czechoslovakia and Italy.

Multiple methods were used to investigate attitudes and behaviours of GPs, these included physician questionnaires (n = 9), patient interviews (n = 5) and questionnaires (n = 4), case-note reviews (n = 3) and physician interviews (n = 1).

### Quality Appraisal

Both quality assessment tools [19,20] highlighted similar strengths and weakness of the studies. Disagreements between assessors occurred in 8% of initial decisions and all were resolved. A summary of the agreed quality assessment results, using the NCAW, are provided in Additional File 3 and further details can be found in Additional Files 1 and 2.

All articles clearly stated the research question, study type and outcome factors. Most articles failed to provide details of ethical approval, and whilst most described their sampling frame, many used small sample sizes of specialist groups or volunteers in limited geographical areas.

Most studies had low response and/or follow-up rates and were therefore open to response bias. Response rates ranged from 7.4%-94% for the studies examining GPs' attitudes towards exercise for KOA and from 27-94% for studies investigating behaviour. Seven of the fifteen relevant studies (47%) had a response rate lower than 50%. Few studies explored the extent and/or likelihood of non-response bias.

Study methods potentially introduced problems, for example, use of lists/multiple-choice response options or structured questionnaires may promote over-reporting of actual behaviours. Recall bias is also inherent in any study relying on patient report.

Studies often failed to discuss the researcher-participant relationship, or how the study tools were developed. The clinical utility of one study [31] was limited by the question posed, "do you provide or refer for the following treatments?" This questioning style attempts to assess whether GPs ever used certain treatments and fails to provide meaningful insight into regular practice.

### Attitudes and Beliefs Concerning Exercise

Of the seven studies reporting attitudes of physicians towards exercise for KOA five used physician-completed questionnaires [24-27,29], one used patient interviews [28] and the other physician interviews [23]. Of these, three directly investigated the attitudes of GPs [23,26,29] but two of these studies focussed on attitudes towards guidelines recommending exercise for KOA [26,29]. Of the remaining four studies, attitudes of GPs were indirectly gained from patient interviews [28] or were implied; as GPs suggested rest, rather than exercise [24,25,27].

A wide range of attitudes of GPs towards exercise for KOA was highlighted, from GPs believing exercise should not be used i.e. they advised rest [24,27], to almost total agreement with guideline recommendations for the use of exercise for KOA [26] [Additional File 1]. Of the seven studies that investigated attitudes and beliefs of GPs towards exercise for KOA, three implied less than positive attitudes [24,25,27]. Hendry et al [28] used patient report, to highlight positive and negative attitudes towards exercise for KOA although the opinions of GPs were not always clear. de Bock et al [23] detailed GP's positive attitudes about physiotherapy compared to pharmacological therapy.

### Behaviour Concerning Exercise

Within the 18 studies investigating behaviour of GPs regarding exercise for CKP/KOA, eight presented information on "reported behaviours" of GPs [4,24-27,30,31,33]. The remaining 10 studies detailed "actual" behaviour using; patient questionnaires (n = 3) [34,38,39], patient interviews (n = 3) [32,37,40], case-note review (n = 3) [23,35,41] and patient questionnaires and interviews (n = 1) [36]. These studies suggest variable inclusion of exercise by GPs in the management of CKP/KOA [Additional File 2]. Although 99% of GPs reported ever providing advice or referring to a physiotherapist [31], the frequency of actual provision of exercise advice or physiotherapy referral was lower. Estimates of provision of exercise advice and physiotherapy referral were generally higher for vignette-based studies (exercise advice 9%-89% [4,24-27,30];physiotherapy referral 44%-77% [27,30,33]) than reviews of actual practice (exercise advice 5%-52% [23,32,36,40-42]; physiotherapy referral 13-63% [23,34,35,37-40]). Of the studies specifically concentrating on CKP, 18-40% patients had received or been referred for physiotherapy [35,38,40], 44-54% of GPs stated they would refer to physiotherapy and 59-76% stated they would advise on knee joint exercises for such patients [30].

## Discussion

Guideline recommendations emphasise exercise as a core first-line management strategy for CKP/KOA in primary care [15] and the UK Department of Health's 2006 Musculoskeletal Services Framework [43] recognised exercise as beneficial in people with osteoarthritis and thus information should be provided to patients to "promote exercise". A systematic literature review was conducted to investigate the attitudes, beliefs and behaviours of GPs, specifically relating to exercise for CKP/KOA.

### Summary of Results

A paucity of studies investigating attitudes and behaviours of GPs regarding exercise for CKP/KOA was identified. This systematic review identified studies that utilised a range of methods including qualitative and quantitative approaches. This prevented use of a single quality assessment tool. Thus two tools were used for each study and provided similar results. Response rates varied widely (7-94%), but were generally poor with 47% of studies having a response rate lower than 50%. Therefore non-response bias may lead to unrepresentative estimates of broader GP populations. Most studies used descriptive questionnaire or interview methods.

Attitudes and beliefs towards exercise for KOA appear to be diverse and, overall, exercise of any type appears to be under-used, -advised and/or -prescribed by GPs managing CKP/KOA. Although 99% of GPs reported ever providing advice or referring to a physiotherapist [31], the frequency of actual provision of exercise advice or physiotherapy referral was lower (6-63%). The methodology used within the study resulted in further differences in the estimates of provision of exercise advice and physiotherapy referral. Use of vignette-based studies generally yielded higher estimates than reviews of actual practice. Results also differed depending on how physicians were questioned about their behaviour. Some studies asked about the GPs' "ever use" of exercise whilst others asked about specific cases. The former style unsurprisingly yielded higher proportions of GPs referring patients with CKP/KOA to physiotherapy [31]. In studies examining "actual" behaviour there was a higher referral rate to physiotherapy than GP provision of advice to exercise. This may result from uncertainty of GPs about the optimum exercises to advise/prescribe or from time restrictions imposed on GPs' patient consultations.

Inconsistencies and/or ambiguity in methodology, definitions, attitudes, beliefs and behaviours under investigation both hindered direct comparison of results and may partly explain the variability observed. Studies investigating attitudes and beliefs of GPs towards exercise for KOA used undefined, non-specific terms such as "suggest" and "recommend". Only one study [28] acknowledged the management spectrum for CKP/KOA, from advice to exercise through to specific exercise prescription. Terminology describing GPs' behaviours included "provide", "prescribe", "recommend", "instruct" and "advise", however, these terms were not defined. Commonly, definitions of the term "exercise" were missing from papers, thus it could not be determined if "exercise" referred to general aerobic exercise, specific quadriceps strengthening exercises, range of movement exercises or all three [Additional File 2].

### Findings in relation to existing literature and guidance

Individual studies investigating attitudes, beliefs and behaviours of GPs regarding exercise for KOA have commented on the under-use of non-pharmacological treatment modalities and, specifically, exercise. A Canadian study, excluded from this literature review due to uncertainty about the relevance of the sample, found that only 63% of patients with KOA symptoms had ever been recommended to undertake exercise [44]. The results of our review support these findings. The frequency of use of exercise for CKP/KOA appears similar to that for hip [45] and back pain [46]. A USA study reported that only 17% of GPs suggested exercise of any type and only 14% referred patients to physiotherapy for hip pain [45]. Another USA study stated 29% of patients had been "prescribed" exercise by a physician for back pain [46].

The Department of Health's Musculoskeletal Framework [43] describes the roles of GPs as being a "direct route into the NHS" and "gatekeepers for other services". It explicitly describes education resulting in reduced prescribing of non-steroidal anti-inflammatory drugs and that GPs undertake a large number of joint injections, however it does not mention the role of GPs in providing exercise advice or prescriptions. Reference within the Framework to the primary care *team *may result in the locus of responsibility for exercise prescription and/or initiation being shifted away from the GPs themselves and on to allied healthcare professionals. These factors may result in varied attitudes and behaviours of GPs depending upon their local service configuration and on their interpretation of their responsibilities within the national Framework. The Framework's Hip and Knee Pain flow chart describes "active management", "facilitate self-management" and "give patient information" as roles of GPs, however, which indicates that GPs should be providing the recommended information on exercise for such conditions.

### Limitations of this study

The key limitation of this systematic review is the paucity of studies found. This indicates that the review topic is relatively under-researched. Few studies described CKP as per our working definition, although many studied KOA, which usually follows a chronic course.

The methodologies used within the included studies may introduce some inaccuracy, for example, it seems that studies that solely relied on GP self-report may have over-estimated exercise behaviours. Further, reliance on patient report of GP behaviours may result in recall bias and under-reporting of behaviours, and record review is only as accurate as the notes made and thus may result in under-reporting of behaviours. Our use of implied beliefs, obtained through extraction of study data may have further skewed data if the physicians' advice to rest did not always indicate a less than positive attitude towards exercise. Such implied beliefs may be biased by multiple unmeasured influences. The interpretation of the terms "exercise" and "rest" may have been different for the authors and the physicians taking part in the study. A physician may advise "rest" from usual physical activities if these are usually of high intensity or knee straining but not be advising complete rest of the knee. However, this latter point was not the case in the study by Chevalier et al [24] as the proportion of patients provided with "joint sparing advice" decreased as the rates of advice for "strict bed rest" increased. The frequency with which GPs exhibited implied negative attitudes and beliefs about exercise for CKP was low, therefore, by eliminating the studies from which implied attitudes and beliefs were extracted, there is still a spectrum of opinion ranging from the more negative and/or ambivalent approaches such as that exercise is unable to change symptoms [23] and that it will at least be less harmful than alternatives [23] through to positive attitudes that physicians generally agree with the use of exercise [29] and almost total agreement with recommendations that include exercise for CKP/KOA [26].

No studies examined the explanations underlying the reported attitudes so it is difficult to draw strong conclusions from the data regarding attitudes. However, published discussions suggest factors that may negatively affect GP's attitudes: exposure to contradictory information [26], concern about lack of efficacy [23] and potential for harm [27].

### Clinical and research implications resulting from this study

Small response rates and use of specialist groups of GPs limited the generalisability of the results of many studies. Such samples may provide over-estimates of exercise behaviour. Given the apparent under-use of exercise by GPs for patients with CKP/KOA, it is possible that the true pattern of practice is even further from exercise recommendations in available guidelines. The negative clinical effect of this apparent under-use of exercise use may be further exaggerated if patients are unable to translate advice or instructions into correctly executed and frequently performed exercises. Dexter et al [32] noted that of those that had been advised to exercise for hip and/or knee OA only 63% did so. In addition, only 10% of patients who were undertaking strengthening and/or stretching exercises of the hip or knee were performing these correctly and regularly.

Individual studies suggested potential reasons for the apparent under-use of exercise by GPs. These include, uncertainty about the role of GPs in relation to exercise for CKP [47,48] and/or appropriate types [36] of exercise, uncertainty of the correct exercise "prescription" [14,47]; lack of awareness about the guidelines [26]; the belief that patients will not exercise [49]; the presence of comorbidities [48]; increasing patient age [47,48]; and limited access to services [50]. Barriers imposed by healthcare systems such as unclear referral criteria, limited onward referral to other healthcare professionals and limited consultation time may prevent GPs from providing their desired management. Østbye et al [51] reinforced the latter issue by identifying that provision of comprehensive management for ten common chronic diseases, including arthritis, exceeds the total time GPs have for all patient care [51]. Future research should focus on consistent investigation of attitudes, beliefs and behaviours of GPs regarding the use of exercise for CKP. Research should identify, or confirm suggested, barriers to the use of exercise for CKP and thus full implementation of national guidelines. System barriers and GP attitudes and behaviours, may vary within and between countries due to local and national differences in healthcare provision. Therefore, further research should utilise large, nationally representative samples of GPs.

The role of GPs in initiating exercise for CKP/KOA was not outlined in studies or guidelines, including the recent NICE guidelines [5,15]. Primary care guidelines recommend "exercise" as a core management approach for CKP/KOA [15] but provide no explicit expectations about whether GPs should *refer *patients for exercise therapies, *advise *general or specific exercises, or *prescribe *exercises. The expected roles of GPs in initiating and supporting exercise in patients with CKP thus requires clarification. Work must also identify the optimal means of supporting and educating GPs at the clinical, educational and service level, to improve certainty and confidence about the value of exercise and to use the exercise recommendations in practice.

## Conclusions

Our systematic review has highlighted a paucity of studies investigating, and variability in, the attitudes, beliefs and behaviours of GPs regarding the use of exercise for CKP. However, this treatment modality appears to be underused by GPs. Future work should investigate the attitudes, beliefs and behaviours of GPs regarding exercise for CKP and clarify the expected roles of GPs to help support the translation of best practice recommendations into everyday clinical care.

## Abbreviations

CASP: Critical Appraisal Skills Programme; CKP: chronic knee pain; GP: general practitioner; KOA: knee osteoarthritis; NCAW: Newcastle Critical Appraisal Worksheet; NICE: National Institute for Health and Clinical Excellence; PCP: primary care physician

## Competing interests

The authors declare that they have no competing interests.

## Authors' contributions

EC carried out the literature search, quality assessment and data extraction. ER and NC assessed identified literature for eligibility, confirmed accurate and consistent data extraction and undertook quality assessment. All authors participated in the design of the study and helped to draft the manuscript. All authors read and approved the final manuscript.

## Pre-publication history

The pre-publication history for this paper can be accessed here:

http://www.biomedcentral.com/1471-2296/11/4/prepub

## Supplementary Material

Additional file 1 **Summary of studies investigating the attitudes and beliefs of GPs towards exercise for KOA**. Table detailing the studies that were included in the literature review that investigated the attitudes and beliefs of GPs towards exercise for knee osteoarthritis including information on the study population, study method, type of exercise under investigation, a summary of the findings and limitations to the quality of the paper and further comments on the paper Click here for file

Additional file 2 **Summary of studies investigating the behaviours of GPs towards exercise for CKP/KOA**. Table detailing the studies that were included in the literature review that investigated the behaviours of GPs towards exercise for CKP/KOA including information on the study population, study method, type of exercise under investigation, a summary of the findings and limitations to the quality of the paper and further comments on the paper. Click here for file

Additional file 3 **Quality appraisal of papers found using The NCAW**. Table summarising the points of The Newcastle Critical Appraisal Worksheet that each study met or did not meet during quality assessment following assessment by two independent assessors with resolution of any disagreements occurring through the use of a third independent assessor. Click here for file
