# Supplementary material for: The attitudes, beliefs and behaviours of GPs regarding exercise for chronic knee pain: a systematic review
Source: BMC Fam Pract. 2010 Jan 18;11:4. doi: 10.1186/1471-2296-11-4 (PMC2826301; doi:10.1186/1471-2296-11-4)
Supplement: Additional file 1 — Summary of studies investigating the attitudes and beliefs of GPs towards exercise for KOA. Table detailing the studies that were included in the literature review that investigated the attitudes and beliefs of GPs towards exercise for knee osteoarthritis including information on the study population, study method, type of exercise under investigation, a summary of the findings and limitations to the quality of the paper and further comments on the paper [file 1471-2296-11-4-S1.DOC]

## Summary of studies investigating the attitudes and beliefs of GPs towards exercise for KOA

| **Study** | **Study Population (response rate)** | Study Method | Type of exercise under investigation* | Findings* | Limitations to quality of the paper | Comments |
| --- | --- | --- | --- | --- | --- | --- |
| de Bock GH et al 1992 [23] | Following record review, there was an investigation of policies of 14 PCPs in the Leiden area, Netherlands (71%) | Semi-structured physician interview | “Referral…to a physical therapist”  “Provided patient information…patient education” (which included education on exercise) | “Physical therapy is less harmful than medical therapy”  “[physical therapy is] unable to change the osteoarthritic symptoms”  “different Dutch PCPs have different policies…in one PCP there is a variation in policy not dependent on the patients’ symptoms…the very divergent rationales and attitudes of PCPs result in very divergent policies” | Lack of consideration of confounding factors, a small sample size, lack of information about the way in which patients were selected and how many PCPs were approached to participate and thus a resulting potential for bias |  |
| Chevalier X et al, 2004 [24] | 4000 PCPs across every region in France (75%) | Vignette-based physician questionnaire | “Strict bed rest”  “Exercise and physiotherapy” | 1.8% of PCPs suggested bed rest for mild OA symptoms, 10% suggested bed rest for moderate OA symptoms and 24% suggested bed rest for severe symptoms  This ***may indicate*** an implied negative attitude towards exercise for KOA and/or the belief that exercise may be harmful. | Use of multiple-choice questions may over-estimate frequency of use of management strategies in actual clinical practice. Confounding factors were not considered, limited information about the sampling method and use of a drug company to recruit physicians may all lead to potential sources of bias. | Vignettes used mechanical pain ***without*** acute exacerbation for the mild and moderate stages of OA symptoms and ***with*** an acute exacerbation in the severe stage |
| Coyte et al, 1996 [25] | 250 randomly selected Family Practitioners from Ontario, Canada (51.6%) | Physician questionnaire | “Prescribe or instruct in physical therapy” | 1% family practitioners stated that they “never or rarely” initiated physical therapy for patients with severe KOA.  This ***may indicate*** a negative attitude towards exercise for severe KOA. | Use of Likert scale may overestimate actual clinical practice. Potential for bias was introduced as a result of confounding factors not being considered, use of only active members of the Ontario College of Family Practitioners and, although no differences were found between responders and non-responders in key demographics, there was only a 52% response rate. |  |
| Denoeud L et al, 2005 [26] | 1030 PCPs randomly selected from database of all PCPs in France (94%) | Physician questionnaire | “Optimal management of KOA requires a combination of pharmacological and non-pharmacological treatment modalities. Non-pharmacological treatment of KOA should include regular education, exercise, appliances and weight reduction” | 99% (n = 954) agreed with the recommendation that includes the use of exercise for KOA and 97% (n = 924) reported that they intended to apply this recommendation. | Lack of consideration for confounding factors may result in the potential for bias | Of those who didn’t intend to apply the recommendations the reasons given included “recommendations did not consider the patient’s opinion”, “too rigid”, thought they “contraindicated information provided by pharmaceutical industry”. |
| Glazier RH et al, 1998 [27] | 775 eligible family physicians from sample of 798 active Ontario members of the College of Family Physicians of Canada (68.3%) | Vignette-based physician questionnaire | “Recommend exercises”  “Recommend rest”  “Referrals physiotherapy” | 29% recommended rest for KOA.  This ***may indicate*** an implied negative attitude towards exercise for KOA and/or the belief that exercise may be harmful. | Potential for over-reporting of actual behaviour through use of multiple-choice management options. Risk of bias introduced by lack of consideration of confounding factors and because differences were found between non-respondents and respondents in relation to their likelihood of being certificants of the College of Family Physicians of Canada. No information was given on statistical analyses. |  |
| Hendry M et al, 2006 [28] | 22 primary care patients with a diagnosis of OA knee from 5 general practices from North Wales, UK (response rate not applicable as qualitative study) | Patient interview | “Exercise was broadly defined to include attending a gym, brisk walking, cycling or participating in sports as well as ‘therapeutic exercises’ prescribed by a health professional.” | “Exercise advice – advice from health professionals was mainly in favour of exercise and consisted of encouragement to exercise, advice about specific exercises and referral to a gym. Sometimes the advice was vague or absent.”  “Occasionally exercise was discouraged” with a patient reporting a “[hospital doctor] said ‘the walking’s agitating your, your joints, so stop it” and “at the hospital they told me I shouldn’t overdo exercise, I should look after my knees”. These findings ***may indicate*** a range of implied attitudes towards exercise for KOA from positive to negative. | Potential for bias introduced through sampling method including recruitment through gyms, use of a limited geographical area (although a range of practices were used to represent a variety of geographic and socioeconomic circumstances) and use of a small sample size. |  |
| Mazieres B et al, 2005 [29] | 30,000 general practitioners in Belgium, France, Italy, Spain and Switzerland (7.4%) | Physician questionnaire | PCPs asked to express their use of “exercise” as a treatment modality  PCPs asked to express the extent to which they agree with the EULAR recommendation “exercises, especially those directed towards increasing strength of quadriceps and/or preserving normal mobility of the knee, are strongly recommended” | For exercise PCPs gave a mean score of 77/100 [SD 21] where 0 = I do not recommend its use and 100 = I do recommend its use. PCPs gave a mean score of 84/100 [SD 16] in agreement with the EULAR guidance to recommend exercise. | Potential for bias introduced as confounding factors not discussed, the samples in each country were recruited through scientific societies and there was a low response rate. |  |

*Direct quotes from papers have been placed in quotation marks, these may be examples of attitudes or beliefs that have been implied or they demonstrate the exact wording used in the study

** Data of implied belief extracted from information given in study thus may be open to inaccuracy

CKP = chronic knee pain; GP = general practitioner; KOA = knee osteoarthritis; PCPs = primary care physicians
