# Supplementary material for: The attitudes, beliefs and behaviours of GPs regarding exercise for chronic knee pain: a systematic review
Source: BMC Fam Pract. 2010 Jan 18;11:4. doi: 10.1186/1471-2296-11-4 (PMC2826301; doi:10.1186/1471-2296-11-4)
Supplement: Additional file 2 — Summary of studies investigating the behaviours of GPs towards exercise for CKP/KOA. Table detailing the studies that were included in the literature review that investigated the behaviours of GPs towards exercise for CKP/KOA including information on the study population, study method, type of exercise under investigation, a summary of the findings and limitations to the quality of the paper and further comments on the paper. [file 1471-2296-11-4-S2.DOC]

## Summary of studies investigating the behaviours of GPs towards exercise for CKP/KOA

| **Study** | **Study Population (response rate)** | Study Method | Type of exercise under investigation* | Findings* | Limitations to quality of the paper | Comments |
| --- | --- | --- | --- | --- | --- | --- |
| ***Studies investigating physician behaviour regarding patients with CKP*** | | | | | | |
| Bedson J et al, 2003 [30] | 1000 randomly chosen full or part-time PCPs in England and Wales (46%) | Vignette-based physician questionnaire | “Advice on knee joint exercises”  Referral to “physiotherapy” | 54% stated they would refer to “physiotherapy” and 59% stated they would provide “advice on knee joint exercises”. | Poor response rate as there was likely to be a difference between responders and non-responders and thus potential for response bias. | The definition of CKP used was chronic knee pain in the absence of joint stiffness, crepitus, soft tissue swelling and quadriceps weakness |
| Linsell L et al, 2005 [35] | Patient records from 604 PCPs in 198 practices across the UK who use the MediPlus database. Records of 3152 patients who presented with new knee pain underwent 36 months follow up (not applicable) | Prospective analysis of records in a PCP database | “Physiotherapy” | 17.7% had received physiotherapy at 36 months | Potential for bias introduced as confounding factors not discussed. | Response rate not applicable as all patients meeting inclusion and exclusion criteria were included |
| Porcheret et al, 2007 [40] | Adults aged >50 years with knee pain who responded to a postal survey of knee pain within 2 general practices in North Staffordshire, UK (36.2%) | Patient interview | “Exercise (excluding advice by physio)”  “Physiotherapy referral” | 46% advised exercise  40% referred to physiotherapy  For both of the above, the proportion advised to exercise or referred to physiotherapy by their PCP cannot be determined. | Limited generalisability as practices used were teaching practices. Potential for responder bias introduced due to low response rate. |  |
| Mitchell HL et al, 2006 [38] | Patients in 2 southeast London PCP practices, UK. (34% of which 49% had knee pain) | Patient questionnaire | “Referred to physiotherapy” | 21% referred for physiotherapy | Potential for bias introduced due to failure to consider confounding factors and the low response rate achieved  Generalisability limited due to limited geographical area used |  |
| ***Studies investigating physician behaviour regarding patients with KOA*** | | | | | | |
| Bedson J et al, 2003 [30] | 1000 randomly chosen full or part-time PCPs in England and Wales (46%) | Vignette-based physician questionnaire | “Advice on knee joint exercises”  Referral to “physiotherapy” | 44-48% stated they would refer to “physiotherapy” and 66-76% stated they would provide “advice on knee joint exercises”. | Poor response rate as there was likely to be a difference between responders and non-responders and thus potential for response bias. | The definition of KOA used was chronic knee pain associated with joint stiffness, crepitus, soft tissue swelling and quadriceps weakness |
| de Bock GH et al 1992 [23] | 196 records of patients with distal osteoarthritis (93 had KOA) randomly selected by 14 PCPs in the Leiden area, Netherlands (71%) | Questionnaire completed through record review and semi-structured physician interview | “Referral…to a physical therapist”  “Provided patient information…patient education” (which included education on exercise) | In 63% cases GPs referred patients to physiotherapy  Patient education was given in 32% cases. | Lack of consideration of confounding factors, a small sample size, lack of information about the way in which patients were selected and how many GPs were approached to participate and thus a resulting potential for bias |  |
| Chard J et al, 2002 [31] | 400 PCPs in UK in association with Primary Care Rheumatology Society (27%) | Physician questionnaire | Provision of or referral to “physiotherapy” | 99% stated they *would* provide or refer for physiotherapy | The nature of the questions used ( “do you ever provide or refer…”) may not have provided a clear picture of regular clinical activity and thus limits the usefulness of the findings. Confounding factors were not considered, the participants belonged to a specialised group and there was a low response rate, all of these factors may lead to bias. |  |
| Chevalier X et al, 2004 [24] | 4000 PCPs across every region in France (75%) | Vignette-based physician questionnaire | “Strict bed rest”  “Exercise and physiotherapy” | 9% stated they use exercise as first line treatment for mild knee OA  Under 15% stated they use exercise as a first line treatment for knee OA of any severity | Use of multiple-choice questions may over-estimate frequency of use of management strategies in actual clinical practice. Confounding factors were not considered, limited information about the sampling method and use of a drug company to recruit physicians may all lead to potential sources of bias. | Vignettes used mechanical pain ***without*** acute exacerbation for the mild and moderate stages of OA symptoms and ***with*** an acute exacerbation in the severe stage |
| Coyte et al, 1996 [25] | 250 randomly selected Family Practitioners from Ontario, Canada (51.6%) | Physician questionnaire | “Prescribe or instruct in physical therapy” | 88.8% family practitioners stated they “often or always” initiate physical therapy for patients with severe KOA, 10.2% stated they “sometimes” do this. | Use of Likert scale may overestimate actual clinical practice. Potential for bias was introduced as a result of confounding factors not being considered, use of only active members of the Ontario College of Family Practitioners and, although no differences were found between responders and non-responders in key demographics, there was only a 52% response rate. |  |
| Denoeud L et al, 2005 [26] | 1030 PCPs randomly selected from database of all PCPs in France were asked to provide details about their management of the first three consecutive outpatients with painful KOA to consult them within an eight week period (94%) | Physician questionnaire | “Prescribed…physical exercise” | 48.7% “prescribed” exercise | Lack of consideration for confounding factors may result in the potential for bias |  |
| Dexter PA et al 1993 [32] | 120 patients with hip and/or KOA recruited from 13 apartment complexes housing elderly persons, Midwestern city, USA (92%) | Patient interview | “Ever received a recommendation from a physician for knee…exercises” | 42% of the total group of patients and 49% of the patients currently seeing a physician for a joint problem, recalled ever receiving medical advice to exercise.  26% patients who had only seen an internist or PCP, remembered receiving a recommendation for exercise | Potential for bias introduced by use of participants from a limited geographical area and lack of information on the total number of patients screened and invited to participate. |  |
| Glazier RH et al, 1998 [27] | 775 eligible family physicians from sample of 798 active Ontario members of the College of Family Physicians of Canada (68.3%) | Vignette-based physician questionnaire | “Recommend exercises”  “Recommend rest”  “Referrals physiotherapy” | 33.1% recommended exercises for KOA  54.2% referred to physiotherapy | Potential for over-reporting of actual behaviour through use of multiple-choice management options. Risk of bias introduced by lack of consideration of confounding factors and because differences were found between non-respondents and respondents in relation to their likelihood of being certificants of the College of Family Physicians of Canada. No information was given on statistical analyses. |  |
| Günaydin I et al, 1997 [33] | 252 general practitioners were selected at random in Baden-Wuerttemberg, Germany (44%) | Vignette-based physician questionnaire | “Physiotherapy” | 77% stated they would refer a patient with KOA for physiotherapy. | Potential for bias introduced through use of a limited geographical are and lack of information provided about non-responders. |  |
| Jordon KM et al, 2004 [34] | 828 patients with diagnosis of KOA and >55yr from two PCP practices in Wessex, UK (56%) | Patient questionnaire | “Hospital physiotherapist”  “Community physiotherapist” | 13% had received physiotherapy from either hospital or GP referrals. The proportion of consultant and/or PCP referrals to physiotherapy could not be determined. | Potential for limited generalisability introduced as both practices used had on-site physiotherapy assessment and direct access to hospital physiotherapy services. Potential for bias as only moderate response rate and non-responders were found to be older than responders. |  |
| Mamlin et al, 1998 [4] | Randomly selected family physicians in Indiana, USA (32.7%) | Physician questionnaire | “Prescribe or instruct in physical therapy” | PCPs prescribed or instructed physical therapy for 25% of patients with severe KOA | Potential for over-reporting of actual behaviour through use of multiple-choice management options. Risk of bias introduced by lack of consideration of confounding factors and poor response rate (although authors report no difference in responders vs non-responders in key demographics) | Physicians were asked to report a percentage of patients for whom they recommended a particular therapy and the mean percentages were recorded |
| Mazzuca SA et al, 1997 [36] | 419 patients looked after by rheumatologist or primary care physician in Indiana, USA (response rate not applicable as taken from patient sample for larger study) | Patient questionnaire, physical examination and interview | “Aerobic activity (e.g. walking)”  “Isometric quadriceps (strength)”  “Range of motion (flexibility)” | 52% advised aerobic activity by PCP  19% advised range of motion by PCP  12% advised isometric quadriceps by PCP | Potential for selection bias introduced as sample recruited by using family physicians who volunteered to assist patient recruitment and thus may be more likely to be interested in KOA |  |
| McHugh GA et al, 2007 [37] | 160 participants identified through recruitment from a hip or knee joint replacement waiting list in a regional orthopaedic centre, UK. (66%) | Patient interview | “Physiotherapy treatments” | 56% people waiting knee replacement had been referred to a physiotherapist by their PCP** | Potential for bias introduced as confounding factors not considered and a third of eligible patients did not respond to initial invitation to participate. Generalisability may be reduced through the use of a limited geographical area and concentration on patients with severe disease only |  |
| Pavelka K et al, 1995 [39] | 137 patients in Czechoslovakia with KOA completed a questionnaire detailing their symptoms and management (response rate not clear as number invited not given) | Patient questionnaire | Uncertain | 62% patients of a group of 20 doctors, consisting mainly of general practitioners, received physical treatment | Information is missing on how the authors collected/recorded data on treatment modalities used and on the sampling method. Potential for bias could not be quantified as no information was given on response rates. | Authors state in the introduction that physiotherapy involves exercises to increase strength of quadriceps and hamstrings |
| Sarzi-Puttini P et al [41], 2005 and Scarpa R et al 2005 [42] | 30,529 patients were enrolled by PCPs, rheumatologists and orthopaedic surgeons in Italy. 12,827 fulfilled criteria and had knee pain. | Record review | “Exercises” | 6% “prescribed” exercise by PCPs | Insufficient data regarding the study type, outcome factors, sampling methods and response rates were provided (although information could be found in accompanying papers). Potential for bias to be introduced as confounding factors not considered. |  |

*Direct quotes from papers have been placed in quotation marks, these may be examples of attitudes or beliefs that have been implied or they demonstrate the exact wording used in the study

**Paper stated 48% of patients awaiting hip or knee replacement were referred to physiotherapy, the figure for those awaiting knee replacement alone were obtained directly from the corresponding author

CKP = chronic knee pain; GP = general practitioner; KOA = knee osteoarthritis; PCPs = primary care physicians
