# Supplementary material for: The attitudes, beliefs and behaviours of GPs regarding exercise for chronic knee pain: a systematic review
Source: BMC Fam Pract. 2010 Jan 18;11:4. doi: 10.1186/1471-2296-11-4 (PMC2826301; doi:10.1186/1471-2296-11-4)
Supplement: Additional file 3 — Quality appraisal of papers found using The NCAW. Table summarising the points of The Newcastle Critical Appraisal Worksheet that each study met or did not meet during quality assessment following assessment by two independent assessors with resolution of any disagreements occurring through the use of a third independent assessor. [file 1471-2296-11-4-S3.DOC]

## Quality appraisal of papers found using The NCAW

| **Item being examined 🡪** | **Research question** | | | **Study type** | | | **Outcome factors** | | | **Confounders** | | | **Sampling** | | | **Internal validity** | | | **Statistical tests** | | | **Significant results (clin/socially)** | | | **Ethical issues** | | | **Conclusions** | | |
| --- | --- | --- | --- | --- | --- | --- | --- | --- | --- | --- | --- | --- | --- | --- | --- | --- | --- | --- | --- | --- | --- | --- | --- | --- | --- | --- | --- | --- | --- | --- |
| **Author 🡫** | **1** | **2** | **3** | **1** | **2** | **3** | **1** | **2** | **3** | **1** | **2** | **3** | **1** | **2** | **3** | **1** | **2** | **3** | **1** | **2** | **3** | **1** | **2** | **3** | **1** | **2** | **3** | **1** | **2** | **3** |
| **Bedson** | Y | N | N | Y | N | N | Y | N | N | N | Y | Y | Y | N | N | Y | Y | Y | Y | N | N | Y | N | N | N | N | N | Y | N | N |
| **De Bock** | Y | N | N | Y | N | N | Y | N | N | N | Y | Y | Y | Y | Y | N | Y | Y | Y | N | N | Y | N | N | N | N | N | Y | Y | Y |
| **Chard** | Y | N | N | Y | N | N | Y | Y | Y | N | Y | Y | Y | Y | Y | Y | Y | Y | Y | N | N | N | Y | Y | N | N | N | N | N | N |
| **Chevalier** | Y | N | N | Y | N | N | Y | Y | Y | N | Y | Y | Y | Y | Y | Y | N | N | N | N | N | Y | N | N | N | N | N | Y | N | N |
| **Coyte** | Y | N | N | Y | N | N | Y | Y | Y | N | Y | Y | Y | Y | Y | Y | Y | Y | Y | N | N | Y | N | N | Y | N | N | Y | N | N |
| **Denoeud** | Y | N | N | Y | N | N | Y | N | N | N | Y | Y | Y | N | N | Y | N | N | Y | N | N | Y | N | N | N | N | N | Y | N | N |
| **Dexter** | Y | N | N | Y | N | N | Y | N | N | Y | N | N | Y | Y | Y | Y | Y | Y | Y | N | N | Y | N | N | N | N | N | Y | N | N |
| **Glazier** | Y | N | N | Y | N | N | Y | Y | Y | N | Y | Y | Y | N | N | Y | N | Y | Y | Y | Y | Y | N | N | N | N | N | Y | N | N |
| **Günaydin** | Y | N | N | Y | N | N | Y | N | N | Y | N | N | Y | Y | Y | Y | Y | Y | Y | N | N | Y | N | N | N | N | N | Y | N | N |
| **Hendry** | Y | N | N | Y | N | N | N/A | N | N | N/A | N | N | Y | Y | Y | N/A | N | N | N/A | N | N | Y | N | N | Y | N | N | Y | N | N |
| **Jordon** | Y | N | N | Y | N | N | Y | N | N | Y | N | N | Y | Y | Y | Y | Y | Y | Y | N | N | Y | N | N | Y | N | N | Y | N | N |
| **Linsell** | Y | N | N | Y | N | N | Y | N | N | N | Y | Y | Y | N | N | Y | N | N | Y | N | N | Y | N | N | Y | N | N | Y | N | N |
| **Mamlin** | Y | N | N | Y | N | N | Y | Y | Y | N | Y | Y | Y | N | N | Y | Y | Y | Y | N | N | Y | N | N | N | N | N | Y | N | N |
| **Mazieres** | Y | N | N | Y | N | N | Y | N | N | N | Y | Y | Y | Y | Y | Y | Y | Y | Y | N | N | Y | N | N | N | N | N | Y | N | N |
| **Mazzuca** | Y | N | N | Y | N | N | Y | N | N | Y | N | N | Y | Y | Y | Y | Y | Y | Y | N | N | Y | N | N | N | N | N | Y | N | N |
| **McHugh** | Y | N | N | Y | N | N | Y | N | N | N | Y | Y | Y | Y | Y | Y | Y | Y | Y | N | N | Y | N | N | Y | N | N | Y | N | N |
| **Mitchell** | Y | N | N | Y | N | N | Y | N | N | N | Y | Y | Y | Y | Y | Y | Y | Y | Y | N | N | Y | N | N | Y | N | N | Y | N | N |
| **Pavelka** | Y | N | N | Y | N | N | Y | Y | Y | Y | N | N | Y | Y | Y | N | Y | Y | Y | N | N | Y | N | N | N | N | N | Y | N | N |
| **Porcheret** | Y | N | N | Y | N | N | Y | N | N | N | N | N | Y | Y | Y | Y | Y | Y | Y | N | N | Y | N | N | Y | N | N | Y | N | N |
| **Sarzi-Puttini** | Y | N | N | Y | N | Y | Y | Y | Y | N | Y | Y | N | Y | Y | N | Y | Y | N | Y | Y | Y | N | N | N | N | N | Y | N | N |
| **For each aspect of the quality appraisal three questions were asked, each number in the above table corresponds to each question:**   1. **Can you find this information in the paper? 2. Is the way this was done a problem? 3. Does this problem threaten the validity of the study?** | | | | | | | | | | | | | | | | | | | | | | | | | | | | | | |
